# Community trust of government and non-governmental organizations during the 2014-16 Ebola epidemic in Liberia

**DOI:** 10.1371/journal.pntd.0010083

**Published:** 2022-01-27

**Authors:** Ronan F. Arthur, Lily M. Horng, Fatorma K. Bolay, Amos Tandanpolie, John R. Gilstad, Lucy K. Tantum, Stephen P. Luby

**Affiliations:** 1 School of Medicine, Stanford University, Stanford, California, United States of America; 2 National Public Health Institute of Liberia, Monrovia, Liberia; 3 Armed Forces Liberia, Monrovia, Liberia; 4 Uniformed Services University of the Health Sciences, Bethesda, Maryland, United States of America; Fundacao Oswaldo Cruz, BRAZIL

## Abstract

The West African Ebola Virus Disease epidemic of 2014-16 cost more than 11,000 lives. Interventions targeting key behaviors to curb transmission, such as safe funeral practices and reporting and isolating the ill, were initially unsuccessful in a climate of fear, mistrust, and denial. Building trust was eventually recognized as essential to epidemic response and prioritized, and trust was seen to improve toward the end of the epidemic as incidence fell. However, little is understood about how and why trust changed during Ebola, what factors were most influential to community trust, and how different institutions might have been perceived under different levels of exposure to the outbreak. In this large-N household survey conducted in Liberia in 2018, we measured self-reported trust over time retrospectively in three different communities with different exposures to Ebola. We found trust was consistently higher for non-governmental organizations than for the government of Liberia across all time periods. Trust reportedly decreased significantly from the start to the peak of the epidemic in the study site of highest Ebola incidence. This finding, in combination with a negative association found between knowing someone infected and trust of both iNGOs and the government, indicates the experience of Ebola may have itself caused a decline of trust in the community. These results suggest that national governments should aim to establish trust when engaging communities to change behavior during epidemics. Further research on the relationship between trust and epidemics may serve to improve epidemic response efficacy and behavior uptake.

## Author summary

Behavior change was critical to the West African Ebola epidemic response in Liberia. Previous studies show trust in the Liberian government was associated with behavior compliance, while hardships related to Ebola were associated with lower levels of trust. Trust of international non-governmental organizations (iNGOs) was consistently higher than trust in the Liberian government. However, studies have not compared exposed communities to non-exposed communities over time. This study measured trust in the government and trust in iNGOs in three communities with different exposure to the Ebola epidemic. Results corroborate that trust of iNGOs was higher than trust in the government across all five time periods. Trust decreased significantly during the peak of the Ebola crisis, especially in the community with the highest incidence. Individuals who believed that Ebola was real and had high levels of knowledge about Ebola had higher trust than those who did not. Being the frequent witness of Ebola-related events was associated with higher trust in iNGOs, but not in the government. These findings indicate that efforts to improve Ebola awareness and knowledge may positively influence trust, while exposure to the epidemic may reinforce prior mistrust of government institutions.

## Introduction

The 2014–16 Ebola Virus Disease (Ebola) epidemic in West Africa, principally in Guinea, Liberia and Sierra Leone, was the deadliest Ebola epidemic in history, with more than 28,000 reported cases and 11,000 associated deaths [1]. Originating in Guinea in late 2013, the Ebola outbreak spread to Liberia’s northern Lofa County by March of 2014 [2] and from there to the urban Montserrado County by June 2014. In August 2014, locals in the Monrovia slum of West Point looted a health center and forcefully withdrew Ebola patients there. The government responded with an enforced quarantine that turned violent when government forces fired shots into the crowd during a protest, resulting in the death of a teenage boy. The event incited fear, mistrust, and public memories of recent civil war [3].

International response to the epidemic, other than through the few institutions already on the ground in Liberia, was slow to mobilize [4, 5]. The majority of Ebola treatment units (ETUs), for example, were constructed after incidence in Liberia had already begun to decline in September 2014 [1, 6]. Numerous health promotion, risk communication, and psycho-social support interventions by international organizations were likewise launched in August–November 2014 [7]. Thus, actions taken by international organizations late to arrive were likely not the sole driver of Ebola incidence decline.

Indeed, modeling indicates a combination of institutional intervention and individual behavior change helped to finally contain and end the epidemic [7, 8]. In the absence of vaccines and approved therapies, non-pharmaceutical interventions were recommended, including handwashing, notifying Ebola response teams of infected persons, and safe burial practices [9]. Some of these behaviors and practices were widely adopted [10, 11], but a few behaviors recognized for their contribution to ongoing transmission, most notably healthcare provided at home by family members and traditional burial practices, proved more difficult to modify [12, 13]. Early government interventions to interrupt transmission often targeted these behaviors without meaningful dialogue with communities to problem-solve and build trust [14].

Many community members rejected quarantine and safe burial interventions and shunned treatment centers out of fear for the safety of the sick [12, 15]. Medical anthropologists found that healthcare avoidance and traditional burial practices were difficult behaviors to alter in a climate of fear, mistrust, and denial [12, 16]. Some individuals would hide themselves or their loved ones if infected, and, in some communities, there were incidents of violent resistance to community health worker groups [14]. Over time, community trust of government institutions and response efforts were eventually recognized as critical to controlling the epidemic as recommended practices would not be taken up in their absence [17].

Qualitative work has contextualized our understanding of individual trust and fear during the 2014–16 Ebola epidemic, including recent history, tradeoffs, and rational perspectives [12, 18], while quantitative studies have measured associations between trust, behavior, and knowledge. Cross-sectional surveys found higher trust in health institutions was associated with higher compliance with Ebola control measures [10]. Another study found differences in Ebola knowledge, attitudes, and practices between high-incidence communities and low-incidence communities: low-incidence communities, which had less exposure to outbreak response interventions, expressed less knowledge of Ebola and more belief in rumors about Ebola transmission [11].

A household survey conducted in Monrovia, Liberia during the Ebola epidemic found higher levels of trust in international non-governmental organizations (iNGOs), such as the Red Cross, Médecins Sans Frontières (MSF), Partners in Health, and Last Mile Health, than in the Liberian government [10]. Local trust of health authorities may thus be differentiated based on the object of trust. In Liberia, in a post-war, post-colonial context with ongoing national government corruption allegations, health-related trust is likely to be influenced by the historical and modern associations with these actors [3, 19]. Trust may also be associated with social capital, the set of resources available to an individual embedded in their social relations [20]—in other contexts, having good neighborly relations and access to resources through social connections has been associated with greater trust in one’s community [21, 22].

Despite the recognized importance of local trust and behavior change during epidemics, social factors that contribute to disease transmission are still comparatively neglected in the infectious disease dynamics literature [23–26], while the quantitative link between trust and public health outcomes is even less explored [10]. The majority of the quantitative literature on trust and behavior relates to US and UK domestic public health issues, such as for vaccination [27–29] or HIV/AIDS [30]. Few studies have examined trust in low-income country infectious disease contexts [31–33]. In the case of Ebola, mistrust in government was a major obstacle to response [12] as early government actions did not improve trust [14], and, in Sierra Leone, trust in the healthcare system was qualitatively found to have improved after the peak of the epidemic due to the perception of improved health management [18]. Trust in government and iNGOs has been quantitatively associated with behavior change in the 2014–16 West African Ebola epidemic [10] and in the 2018–19 DR Congo Ebola epidemic [33]. However, we lack quantitative studies of how trust changed over time during the Ebola epidemic, or indeed in any epidemic context, and possible drivers of such changes. This understanding would improve our knowledge of how trust is affected by epidemics and by health authorities, which could lead to improved epidemic intervention strategies.

This study investigates trust in national government and trust in iNGOs over time in locations with differing levels of Ebola incidence and exposure to epidemic-related activity in Liberia. We conducted household surveys in three Liberian communities that had different experiences during the 2014–16 Ebola epidemic. We retrospectively measured self-reported levels of trust in the government and iNGOs during five time periods that cover the core time frame of the Ebola epidemic. Here we report differentiated trust over the course of the epidemic, between communities, and between the object of trust, and we begin a hypothesis-generating analysis of potential explanatory variables.

## Materials and methods

### Ethics statement

This protocol was reviewed and approved by Stanford University (Protocol no. 34436) and University of Liberia Institutional Review Board (Protocol no. 17–11-083) committees. Permission to work in Duazon, Careysburg, and Tubmanburg was granted by the Liberian Ministry of Health and local leadership in each of the study locations. Each respondent was verbally guided through an informed consent document and signed their consent. Researchers were instructed to stop the survey should the subject matter prove too emotionally burdensome to continue, though no such incidents were reported.

### Study design

This study is based on a large cross-sectional household survey conducted in three Liberian study sites in January and February of 2018. Survey questions focused on trust in the government and iNGOs at five different time periods during the 2014–16 Ebola epidemic. This study is part of a three-phase, mixed-methods project. Phase 1 includes an initial qualitative exploratory focus group and pilot study which informed study locations, study design, and survey content, Phase 2 includes the household survey analyzed in this manuscript, and Phase 3 includes a series of nine focus groups to qualitatively investigate the lasting effects of Ebola on behavior and trust.

### Study sites

This research took place in three different Liberian communities: Duazon, Margibi County; Careysburg, Montserrado County; and Tubmanburg, Bomi County. These locations were selected purposively for their diverse experiences during the 2014–16 Ebola epidemic. Duazon, a community in Margibi County located near the Armed Forces of Liberia central barracks at Camp Edward Binyah Kesselly, was exposed to significant epidemic-related activity. Duazon had a projected population of 2,629 in 2014 [34]. Duazon is on the road between Roberts International Airport and Monrovia and situated between major hospitals used for Ebola treatment during the epidemic and sites utilized for cremation. Locals reported that ambulances carrying the bodies of deceased Ebola patients would travel between John F. Kennedy Medical Center in Monrovia and the Eternal Love Winning Africa (ELWA) Hospital and sites designated for the cremation of Ebola victims. While no Ebola cases were reported within Duazon, there were cases in nearby communities.

Careysburg in Montserrado County is a remote, rural community with low exposure to the 2014–16 Ebola epidemic. Careysburg had a projected population of 10,525 in 2014 [34]. No cases were reported within city limits, according to the residing mayor at the time of our survey. During the epidemic, community policies were established that prohibited visitors from outside of Careysburg to enter, including family members of residents, and prohibited any resident who left the city from returning until the crisis period was over.

Tubmanburg is the capital of Bomi County, located in the northwest of Liberia, closer to neighboring Ebola-stricken countries and more isolated from the capital than Duazon. The city of Tubmanburg had a projected population of 14,576 in 2014 [34]. During the Ebola epidemic, Tubmanburg had an estimated 250 suspected or confirmed cases by January 2015 and became the site of the first ETU built by the United States government [35].

### Selection and enrollment of respondents

Each of the three study site towns was divided into locally recognized neighborhoods with the assistance of local leadership. Field researchers randomly selected four neighborhoods for each of the study locations and systematically sampled houses using a random walk method [36] from neighborhood centers and a constant interval (see S1 Text in the Supplementary Information for field researcher instructions).

Upon visiting a household, the field team listed all adults living within the household and chose one adult randomly for inclusion using four criteria: age of at least 18 years, a resident of the household being surveyed, a resident of the same community during 2014 and 2015, and of the gender opposite to the previous respondent. All adults meeting the above criteria were then listed, and one was selected at random using numbered cards. If the selected household member was not at home, an appointment was scheduled for the researcher to return to conduct the survey. If the respondent declined to participate, the researcher moved to the next household.

### Study instrument

The questionnaire, written in English and verbally administered in Liberian English, was structured in four sections and took approximately 45 minutes to complete [37]. The first section consisted of 21 questions and addressed enrollment criteria and basic demographic information, such as gender, religion, age, political preferences and other characteristics (Table 1). “No response” was a possible answer choice. In the second section, respondents were asked a series of questions to quantify their social capital. Social capital was assessed using a set of 34 resource generator questions, a method developed to quantify social capital through asking about access to tangible goods or benefits through the individual’s social contacts [38]. A set of these questions (S1 Table) was adapted to Liberian culture and aggregated to produce a social capital index score that could then be utilized to understand the association between social capital and reported levels of trust.

**Table 1:** Descriptive statistics of respondents (N = 1433) in aggregate and in each of three study locations: Duazon (peri-urban, high Ebola exposure), Careysburg (rural, low exposure), and Tubmanburg (urban, high incidence). * indicates value for this region is significantly different (*p* < 0.05) than the other two regions according to a Fisher’s Exact test used pairwise for proportions and a t-test used pairwise for numeric variables.

	All (n = 1433)	Duazon (n = 457)	Careysburg (n = 476)	Tubmanburg (n = 500)
Demographics				
Percent female	52% (748)	49% (224)	54% (226)	53% (258)
Mean age (years)	34.0	33.7	36.2*	32.3
Mean household size	6.15	6.42	6.08	5.97
Percent Christian	81% (1162)	93% (426)*	83% (396)*	68% (340)*
Percent Muslim	11% (156)	2% (11)*	4% (21)*	25% (124)*
Mean social capital index (SD)	49.6 (13.8)	56.9 (14.0)*	44.8 (13.2)*	47.6 (11.4)*
Median (range)	49 (6–88)	58 (18–88)	44 (6–83)	47 (8–83)
Education level (Percent)				
None	17% (245)	12% (57)*	20% (97)	18% (91)
Some primary	16% (224)	18% (80)	17% (81)	13% (63)
Completed primary	16% (226)	17% (76)	17% (82)	14% (68)
Some high school	27% (389)	23% (105)	26% (123)	32% (161)
Completed high school	15% (215)	19% (86)	11% (54)	15% (75)
Some university	7.8% (112)	9.6% (44)	6.3% (30)	7.6% (38)
Completed university	1.5% (22)	2.0% (9)	1.9% (9)	0.8% (4)
Political Party (Percent)				
Congress for Democratic Change (Weah)	71% (1015)	74% (340)	72% (342)	67% (333)
Unity Party (Boakai)	17% (242)	14% (66)	11% (52)	25% (124)*
Neither	11% (162)	10% (47)	16% (74)	8.2% (41)
No response	1.0% (14)	0.9% (4)	1.7% (8)	0.4% (2)

The third section consisted of questions about the respondent’s experience and knowledge of Ebola. Respondents were asked if they knew someone who had been infected with Ebola, how often they saw Ebola-related activity during the epidemic, and 6 questions about how well the respondent felt they could trust information from the following sources during the Ebola epidemic: the radio, the Armed Forces Liberia, community health workers, community leader(s), other friends/contacts in the community, SMS information campaigns. Answers ranged from 1–5 on a 5-point Likert scale as follows: 1—“completely trust”; 2—“somewhat trust”; 3—“neither trust nor distrust”; 4—“somewhat distrust”; 5—“completely distrust”, “no response”. The team asked respondents 6 true-false questions about Ebola and its transmission to assess the respondent’s beliefs about the disease and familiarity with scientific information. These answers were graded against scientific consensus and aggregated to produce a possible score of 0–6. Responses of “I don’t know” were coded as incorrect.

**Fig 1:**
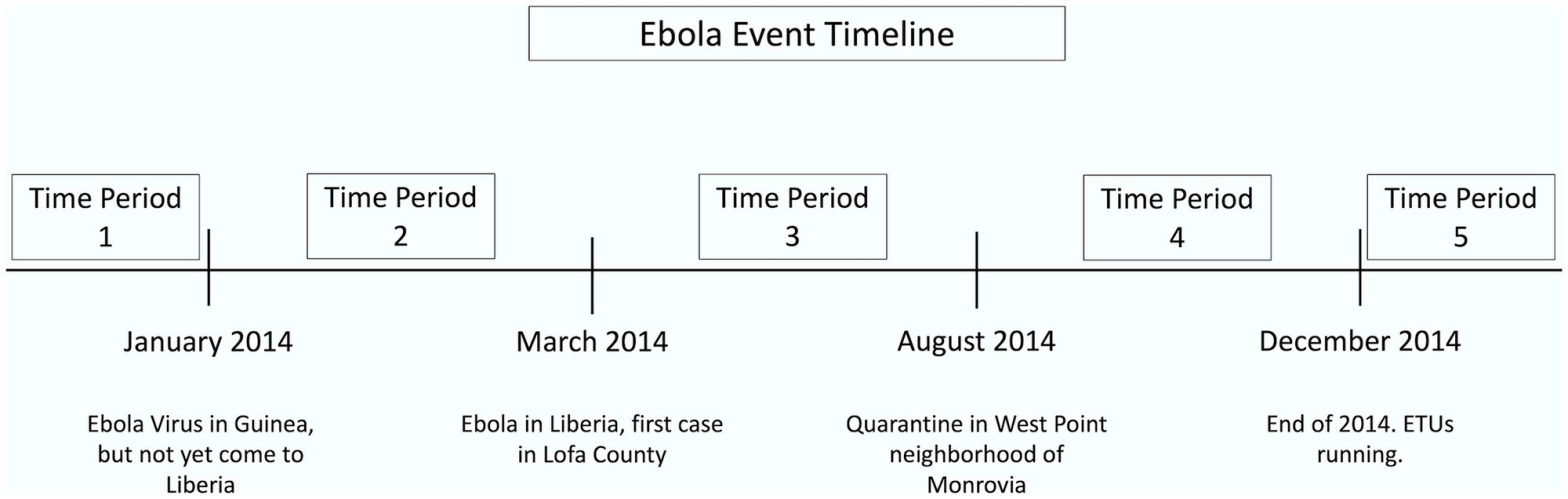
Ebola event timeline defining time periods in relation to recognized events.

The fourth section contained 10 retrospective questions about Ebola-related perceptions of Liberian government and iNGO trustworthiness at different time periods during the epidemic. In this section, researchers used a timeline that visually illustrated the time period scheme in the survey (Fig 1). The researcher guided the respondent through the definition of time periods by using events that all or nearly all adult respondents would recognize as the signposted boundaries of each time period. These boundaries were developed through focus group discussions with Liberian healthcare workers who had lived through the crisis and included memorable Ebola events for Liberians. Researchers piloted questions to determine validity and interpretation of the survey tool prior to implementing the questionnaire at study sites. The respondent’s answer to the question, “Did you trust the Liberian government (President Ellen Johnson Sirleaf’s administration) to protect your health during time period xyz?” was recorded with answers ranging from 1–5 on a 5-point Likert scale as previously described and a 6th category for non-response. This question was repeated for 5 time periods, encompassing the span of the epidemic timeline from 2014–15. To measure trust in iNGOs, which were defined as representatives of foreign aid or health organizations, such as the Red Cross or MSF, researchers asked the same questions with iNGOs as the object, in place of the Liberian government.

### Statistical analysis

Table 1 gives descriptive statistics of the sample population from Section 1 of the survey instrument. Proportions (e.g. proportion of sample with Some primary education) were compared between study sites and tested for significance using Fisher’s Exact Test, run in pairwise combination. Means (e.g. age) were compared between study sites, and the significance of the difference between means was tested using a pairwise t-test (Tables 1 and 2). Trust of government and of iNGOs were compared during each time period and by region (Tables 3–5). Using social capital index scores, the mean and standard deviation of the resulting distributions were calculated, and the means for each study site were compared using pairwise t-tests (Table 2). An ordinal logistic regression model was used with trust as the dependent variable and institution, region, or time period as the independent variables each evaluated separately. Odds ratios and 95% confidence intervals were calculated from the coefficients of these regressions. Missing data were removed, resulting in 1375 responses of the original 1433. To begin a hypothesis-generating analysis of explanatory variables for trust of government and of iNGOs during the peak of the Ebola epidemic, we used an ordinal logistic regression model with the 5-point Likert scale trust responses reported for the government and for iNGOs as the dependent variables. Missing data were removed from the analysis, resulting in 1358 responses from the original 1433. We included the following standardized independent variables: gender (male/female), religion (Christianity/Islam/Other), political affiliation (Unity Party/Congress for Democratic Change/Neither), study site (Duazon/Tubmanburg/Careysburg), mobility, belief that Ebola was real (Yes/No/No response), social capital score, family size, knowledge about Ebola (True/False score), and knew an infected individual (Yes/No/No response). We used R software to calculate and minimize root mean square error (RMSE) and chose the final model to minimize Akaike Information Criterion (AIC) in a stepwise algorithm.

**Table 2:** Ebola trust, experiences, and knowledge in aggregate (n = 1433) and in each of three study locations: Duazon (peri-urban, high Ebola exposure), Careysburg (rural, low incidence), and Tubmanburg (urban, high incidence). Non-response for Ebola knowledge questions were assumed incorrect. * indicates value for this site is statistically significantly different (*p* < 0.05) than the other two sites according to a Fisher’s Exact Test used pairwise for proportions and a t-test used pairwise for numeric values.

	All (n = 1433)	Duazon (n = 457)	Careysburg (n = 476)	Tubmanburg (n = 500)
During Ebola, you could (completely or somewhat) trust information from:				
Radio	84% (1154/1379)	82% (355/435)	80% (367/459)	89% (432/435)
Armed Forces Liberia	88% (814/929)	81% (277/343)	91% (205/226)	92% (332/360)
Community health workers	89% (1240/1387)	84% (359/427)	90% (415/462)	94% (466/498)
Community leader	88% (1226/1396)	84% (360/428)	87% (407/469)	92% (459/499)
Other friends/contacts in the community	84% (1182/1414)	83% (368/443)	82% (387/473)	86% (427/498)
SMS health communication campaign	87% (796/919)	82% (319/390)	90% (224/250)	91% (253/279)
Frequent witness of Ebola events–At least once a day	75% (1075/1427)	68% (310/455)	69% (326/474)	88% (439/498)*
Knew someone infected with Ebola	63% (899/1332)	53% (241/457)	54% (255/475)	81% (403/500)*
Belief that Ebola was real	88% (1240/1411)	87% (389/446)	87% (408/471)	90% (443/494)
Highly mobile– Leaves community more than once a week	64% (911/1422)	67% (306/455)	57% (269/470)*	68% (336/497)
Ebola questions correct mean:	75% (6423/8598)	72% (1974/2742)	75% (2134/2856)	77% (2315/3000)
A dead body can infect others (True)	87% (1240/1433)	76% (349/457)*	88% (417/476)*	95% (474/500)*
Ebola is caused by witchcraft (False)	77% (1104/1433)	72% (349/457)*	88% (417/476)*	95% (474/500)*
Ebola originally came from wild animals (True)	73% (1042/1433)	65% (299/457)	71% (338/476)	81% (405/500)*
Body fluids can contain Ebola (True)	89% (1277/1433)	84% (385/457)	87% (416/476)	95% (476/500)
Ebola can be carried by mosquitos (False)	57% (810/1433)	62% (281/457)	57% (273/476)	51% (256/500)
Ebola can be found in drinking water (False)	66% (950/1433)	73% (332/457)	67% (318/476)	60% (300/500)*
Mean score (SD, Std error)	4.48 (1.22, 0.03)	4.32 (1.39, 0.06)	4.48 (1.22, 0.06)	4.63 (1.03, 0.05)*
Median (range)	5 (0–6)	5 (0–6)	5 (0–6)	5 (2–6)

**Table 3:** Odds ratios and 95% confidence intervals for trust of iNGOs vs. government and trust in Tubmanburg (urban, high incidence) and Careysburg (rural, low exposure) vs. in Duazon (peri-urban, high exposure) at each of five time periods. Odds ratios were calculated from an ordinal logistic regression model. In the first entry, the odds of reporting trust of iNGOs as higher than trust of the government in the first time period were 2.35, holding all other variables constant.

	Institutional trust	Regional trust	
	iNGO vs. government	Tubmanburg vs. Duazon	Careysburg vs. Duazon
Time period	Odds Ratio [95% CI]	Odds Ratio [95% CI]	Odds Ratio [95% CI]
Time period 1	2.35 [2.02,2.72]	2.88 [2.41,3.46]	1.76 [1.47,2.10]
Time period 2	2.87 [2.48,3.34]	2.85 [2.38,3.41]	1.59 [1.34,1.89]
Time period 3	3.21 [2.76,3.72]	2.18 [1.83,2.61]	1.61 [1.35,1.91]
Time period 4	3.01 [2.60,3.50]	2.01 [1.68,2.40]	1.56 [1.31,1.86]
Time period 5	3.26 [2.80,3.80]	2.07 [1.73,2.48]	1.53 [1.28,1.83]

**Table 4:** Odds ratios and 95% confidence intervals for trust in Time Periods 2–5 vs. in Time Period 1 for trust of the government in Duazon (peri-urban, high exposure), Careysburg (rural, low exposure), and Tubmanburg (urban, high incidence). Odds ratios were calculated from an ordinal logistic regression model. In the first entry, respondents of Duazon were 15% (1–0.92) less likely to report a higher trust rating in Time Period 2 as compared to Time Period 1.

	Trust of government		
	Odds Ratio [95% CI]		
Time period	Duazon	Careysburg	Tubmanburg
Time period 2 vs. 1	0.85 [0.67,1.08]	0.74 [0.58,0.94]	0.78 [0.61,0.99]
Time period 3 vs. 1	0.86 [0.67,1.09]	0.74 [0.58,0.94]	0.60 [0.48,0.77]
Time period 4 vs. 1	0.97 [0.76,1.23]	0.83 [0.65,1.05]	0.63 [0.49,0.80]
Time period 5 vs. 1	1.06 [0.83,1.34]	0.90 [0.71,1.14]	0.73[0.57,0.93]

**Table 5:** Odds ratios and 95% confidence intervals for trust in Time Periods 2–5 vs. in Time Period 1 for trust of iNGOs in Duazon (peri-urban, high exposure), Careysburg (rural, low exposure), and Tubmanburg (urban, high incidence). Odds ratios were calculated from an ordinal logistic regression model. In the first entry, respondents of Duazon were 8% (1–0.92) less likely to report a higher trust rating in Time Period 2 as compared to Time Period 1.

	Trust of iNGOs		
	Odds Ratio [95% CI]		
Time period	Duazon	Careysburg	Tubmanburg
Time period 2 vs. 1	0.92 [0.71,1.18]	0.93 [0.71,1.22]	1.15 [0.85,1.56]
Time period 3 vs. 1	1.00 [0.78,1.29]	1.05 [0.80,1.38]	0.99 [0.73,1.34]
Time period 4 vs. 1	1.10 [0.85,1.42]	1.08 [0.82,1.42]	0.97 [0.72,1.32]
Time period 5 vs. 1	1.35 [1.04,1.74]	1.27 [0.96,1.68]	1.19[0.87,1.64]

### Patient and public involvement

In the feasibility stage of research, a focus group of 10 Liberian nurses was held to consult on study design, methods, and implementation. Questionnaire validation, study site selection, and cultural considerations for recruitment were significantly influenced by these discussions. Leadership in each of the three townships provided implementation context and support and will assist in local meaning-making, interpretation, and dissemination of the results back to the communities involved.

### Dryad DOI

10.5061/dryad.h44j0zpmv [37].

## Results

A total of 1,433 participants were surveyed in Duazon (n = 457), the peri-urban site with high exposure to Ebola response, Careysburg (n = 476), the rural low exposure site, and Tubmanburg (n = 500), the urban high incidence site (Table 1). The mean age of the sample population was 34 years (SD = 12, range = 18–92); 52% of all participants were female. Twenty-five percent of Tubmanburg respondents were Muslim, roughly double the national average of 12.2% [34] and significantly higher than the other two sites (*p* < 0.01). Tubmanburg residents were also more likely to support the Unity Party (the party of former president Ellen Johnson Sirleaf) in recent national elections (25%) as compared to respondents in Careysburg and Duazon (*p* < 0.01).

Performance on the Ebola knowledge and beliefs quiz (Table 2) was high across the sample population (mean = 4.48 correct answers out of a possible 6, range = 0–6, SD = 1.22). It was well known that Ebola could be found in body fluids (89% agreed) and that a dead body could still be infectious (87% agreed). Common misconceptions included that Ebola can be carried by mosquitoes (42% agreed), that Ebola can be found in drinking water (33% agreed), and that Ebola is caused by witchcraft (22% agreed). Residents of Tubmanburg, the urban high-incidence study site, had a higher aggregate score on these questions than residents of Duazon, the peri-urban site with high exposure to Ebola response interventions (Tubmanburg mean of 4.63 correct answers vs. Duazon mean of 4.32, *p* < 0.001).

**Fig 2:**
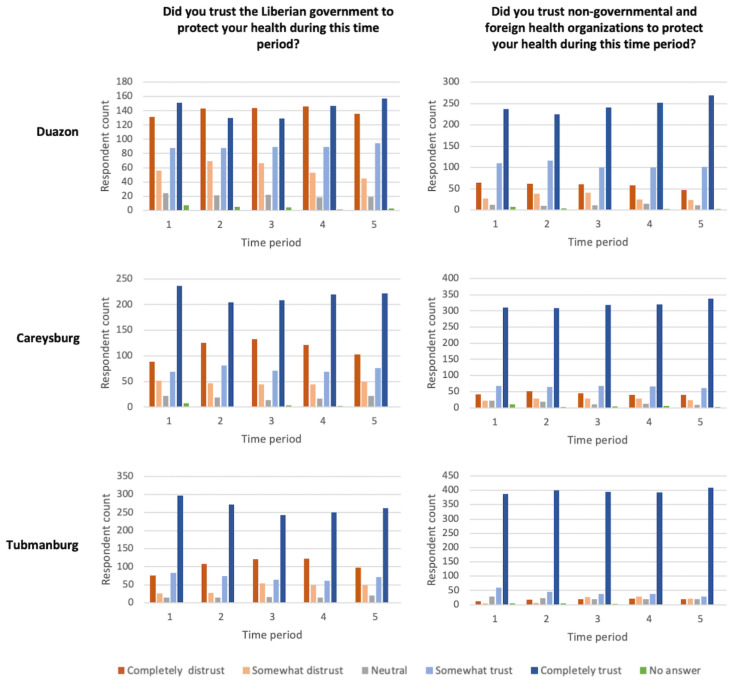
Change over time in trust in government and iNGOs in each of three study locations: Duazon (peri-urban, high Ebola exposure), Careysburg (rural, low exposure), and Tubmanburg (urban, high incidence). Time period 1 = before Ebola came to Liberia, Jan 2014; Time period 2 = between 1st case in Lofa and 1st case in Monrovia; Time period 3 = between 1st case in Monrovia and quarantine in West Point; Time period 4 = between quarantine in West Point and the end of 2014; Time period 5 = beginning of 2015 as last cases of Ebola occurred and schools opened.

Likert-scale trust responses of government and of iNGOs demonstrate differentiation by region and by object of trust over the five time periods (Fig 2). Respondents consistently reported trusting iNGOs to protect their health more than they trusted the Liberian government to protect their health in all five time periods (Table 3). The odds of reporting trust of iNGOs as higher than trust of the government ranged between 2.35 in Time Period 1 to 3.26 in Time Period 5. Overall, trust of government and of iNGOs was higher in Tubmanburg (urban, high incidence) than in the other two study sites, and this differentiation was greatest in Time Period 1.

Trust of government in Tubmanburg, the urban high incidence site, was significantly greater in Time Period 1 compared to Time Periods 2–5, with the greatest difference between Time Periods 1 and 3, for which participants were 40% less likely to rate their trust in government as higher in Time Period 3 than in Time Period 1 (Table 4). Trust of iNGOs was not found to change compared to Time Period 1 for all three regions, except for a small increase in Time Period 5 in Duazon, the peri-urban high exposure site (Table 5).

**Fig 3:**
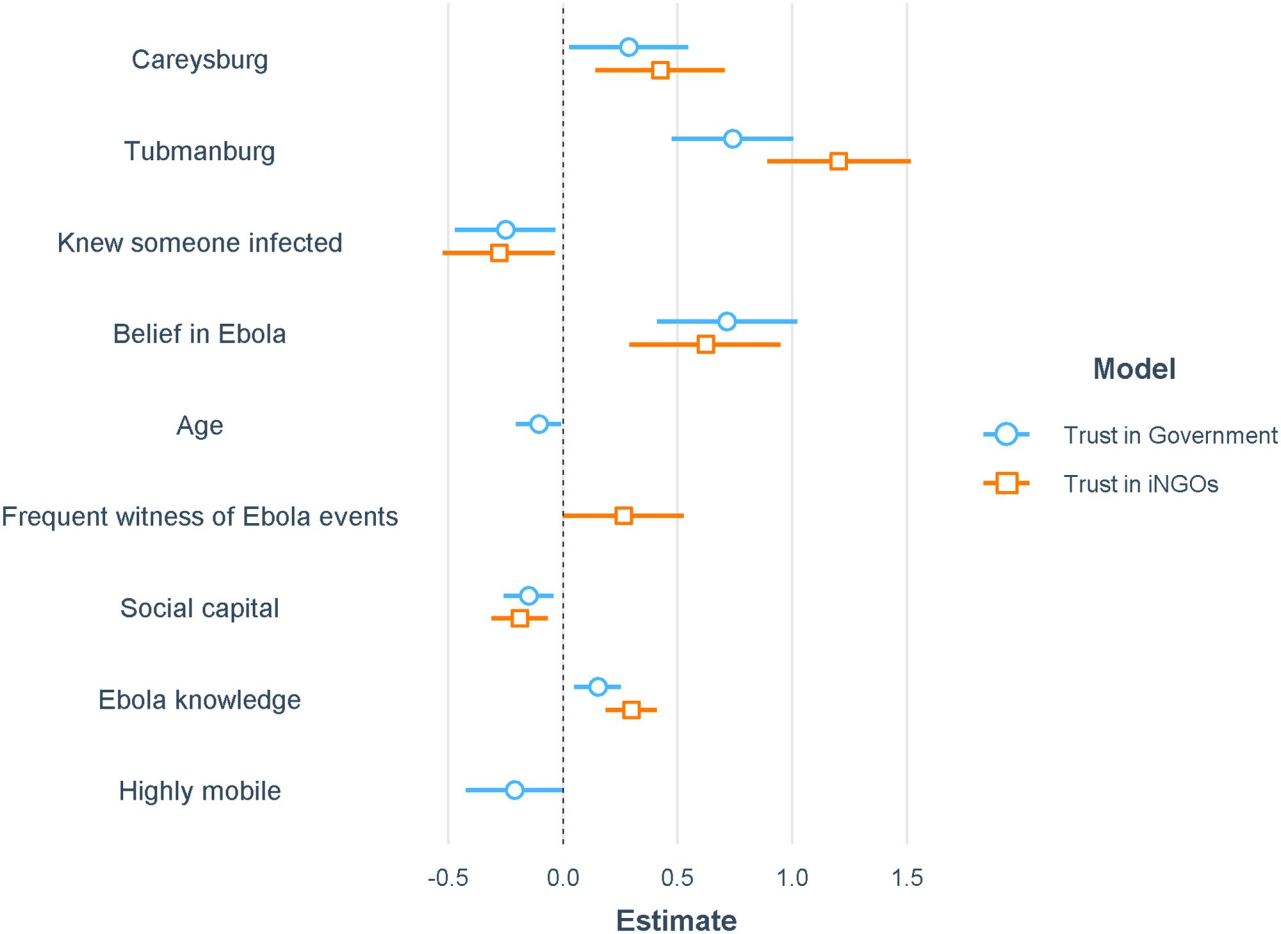
Generalized linear model regression coefficient estimates for trust in government and trust in iNGOs as dependent variables in time period 3 using an ordinal regression model. Some independent variables were dropped via AIC stepwise reduction from one or both models. A negative coefficient indicates a negative association with trust, while a positive coefficient indicates a positive association with trust. Community descriptions: Careysburg = rural, low exposure; Tubmanburg = urban, high incidence.

The belief that Ebola was real, being a resident of the urban high-incidence community of Tubmanburg, being a resident of the rural low-exposure community of Careysburg, and higher knowledge of Ebola were all associated with trust in the Liberian government and trust in iNGOs (Fig 3), according to ordinal logistic regression analysis of trust during Time Period 3, the peak of the epidemic. Frequently witnessing Ebola-related events (at least once a day) was positively associated with trust in iNGOs. Having a relationship with someone infected with Ebola and social capital were negatively associated with trust in the government and in iNGOs, while being highly mobile (leaving the community more than once a week) was negatively associated with trust in the government.

## Discussion

Data on community trust from this large household survey study carried out in Liberia in the aftermath of the 2014–16 Ebola epidemic indicate trust levels, especially of the Liberian government, were distinct in areas with different experiences during the crisis. Respondents were more likely to recall their trust of iNGOs as higher than their trust of the government across all five time periods in agreement with a cross-sectional household survey conducted in Monrovia, Liberia [10]. This result was qualitatively corroborated in focus groups conducted in these communities in a parallel study on post-Ebola era trust [39]. Many respondents in these open discussions drew a distinction between iNGOs and government, perceiving all foreign organizations, including foreign government organizations and iNGO-established Ebola treatment units, to be iNGOs. They perceived government as encompassing any institutions, activities, or representatives sponsored by the Liberian government, including government-run hospitals. Some rationalized the iNGO-government trust discrepancy by explaining that iNGOs had no ulterior motives than simply to help during the crisis, while the government was financially benefiting from the epidemic and was therefore incentivized to prolong the disaster and even to have deliberately caused it in the first place. This may also explain the association between trust in iNGOs and witnessing Ebola-related events frequently, an association which was not found for trust in the government. The a priori trust of each institution and perceived incentives for their epidemic activity may have led to the reinforcement of those perspectives when witnessing Ebola-related activity.

In Tubmanburg, the area of study with the highest Ebola incidence, there was the highest degree of difference between trust in the government between Time Periods 1 and 3, from the beginning stage to the middle stage of the epidemic. This decrease was not replicated in Duazon, the peri-urban high exposure site, but it was replicated to a lesser extent in Careysburg, the rural, low exposure site. Other studies have found that the initial top-down Ebola interventions, such as enforced cremation, quarantines, and bushmeat prohibitions, decreased public trust [14]; hardships experienced in connection with the epidemic decreased trust [10]; and the public feared Ebola treatment units would lead to the death of sick loved ones [12, 39]. During the Ebola epidemic in Guinea and the subsequent epidemic in the Democratic Republic of the Congo, there were accounts of violent actions taken against healthcare teams, and trust overall was reported to be low [40, 41]. Thus, the incidence of Ebola in Tubmanburg may have led to negative experiences with Ebola response and caused the sharp decline in trust found in this study. In Careysburg, there were heavy quarantine restrictions forbidding the entry of foreign or returning persons to the community. Trust in the government to protect community health cannot be divorced from other trust-related perceptions of the government. In the case of Tubmanburg, public trust may be influenced by recent history of brutal civil war, as the city was occupied by a rebel group and experienced a major battle during the war [42]. More broadly, Liberia has experienced ongoing corruption allegations and a stalled economy with few employment opportunities, and is ranked among the poorest economies in the world by GDP per capita [43].

Given the importance of trust to epidemic intervention and the higher trust afforded to iNGOs than to the government, we may conclude that trust of iNGOs may be leveraged to support public health communication and behavior campaigns. However, this conclusion risks exacerbating the alienation of the government from the populace if iNGOs are perceived as more benevolent entities. The relationship between iNGOs and government in weak states, states with fragile institutions and stagnant economies, is complex and can take many forms, at times adversarial or competitive when the viewpoints of the two parties diverge [44]. In the case of epidemics, there is an opportunity for more cooperative relations because the goals of the national government and the iNGOs are largely aligned. Government and iNGOs may achieve goals through different means, undertaking complementary approaches toward a single objective. Closer collaboration may benefit perception of the government, thereby improving behavioral compliance, provided authorities take care to respectfully acknowledge and account for local beliefs, customs, and leadership. At the same time, however, government collaboration may pose a reputational risk for iNGOs, particularly in communities where trust in government is low.

The decline of trust in government public health interventions during the peak of the Ebola epidemic in the community with highest Ebola incidence is concerning because it may indicate that in the absence of effective intervention, a high number of cases may beget more cases not simply due to the nonlinear growth of contagion, but also because of decreased trust. Health economic models which incorporate adaptive behavior change during epidemics typically assume a negative feedback between disease incidence and behavior—as incidence increases, behavioral compliance increases as well, reducing the reproduction number of the epidemic [45–47]. Empirical evidence of this negative feedback relationship has been found in high-income country contexts [48]. However, few studies of this sort have been conducted in low-income country or weak state contexts where low trust may compromise buy-in to behavior change interventions. Fear can drive behavior change, but without perceived response efficacy and confidence of the prescribed behaviors—which in turn requires trust—the set of adopted behaviors may not conform with those that best evidence suggests would actually reduce risk [49, 50]. If increased incidence degrades trust in a weak-state government, as was the case during the Ebola epidemic in Liberia [10], then the relationship between trust and incidence may actually produce a positive feedback of increased transmission and decreased trust, undermining the expected dampening effects of behavior change.

These study results should be interpreted in light of the limits of our methods. We measured self-reported perceptions of trust during different parts of the 2014–15 Ebola epidemic in 2018, subjecting the data to recall bias and bias introduced by socially-reinforced post hoc narratives about the community’s shared experience. While blame-driven narratives may have led respondents to recall a heightened degree of mistrust in government, we consider it more likely that the emotional impact of the epidemic has diminished over time, leading to an underestimation of the effect of epidemic events on trust. Given the time elapsed and the diminishing accuracy of memory, we make no claim that we have accurately measured actual levels of trust over time during the Ebola epidemic, a metric we would have needed to acquire during the epidemic itself. However, these data are still useful as they describe and pertain to the community’s narrative of what happened during the epidemic and still allow for quantitative study of this narrative—differences between groups, time periods, and associations with other variables. Today’s perceptions of trust may inform community actions during the COVID-19 pandemic and future public health emergencies. Furthermore, the consistency of our findings with other studies of trust [10, 12, 18] that deployed somewhat different methods during the Ebola epidemic supports the validity of our primary conclusions.

Ultimately, the Ebola epidemic in West Africa was largely under control by early 2015, but not before claiming the lives of over 11,000 people. The contributing problems associated with mistrust, misinformation, and behavioral non-compliance experienced in Liberia were repeated in the Ebola epidemic in the Democratic Republic of the Congo, beginning in 2018 [33], and in the ongoing coronavirus (COVID-19) pandemic [51]. We may expect such social reactions in future infectious disease outbreaks to recur, and therefore, the international community and national governments should plan accordingly. Specific attention should be paid to weak states where weak health institutions and their associated low levels of trust are often combined with locations of the greatest geographic risk of zoonotic spill over. We recommend further research on trust, including concurrent study of trust during future public health crises [52]. This and other exploratory studies could inform development of community-based interventions to build trust and improve buy-in during response efforts, similar to the interventions put into place during later stages of the Ebola epidemic in Liberia [53]. Trust-building interventions could serve not only to support responses to ongoing disease outbreaks, but also to prevent future epidemics by strengthening community involvement in public health.

## Supporting information

S1 Text Random walk instructions. (PDF)

S1 Table Social capital-related questions and answers for the sample population (n = 1433). (PDF)

S2 Table Generalized linear model regression coefficient estimates for trust in government and trust in iNGOs after AIC stepwise reduction. (PDF)
