# Supplementary material for: Community trust of government and non-governmental organizations during the 2014-16 Ebola epidemic in Liberia
Source: PLoS Negl Trop Dis. 2022 Jan 27;16(1):e0010083. doi: 10.1371/journal.pntd.0010083 (PMC8824372; doi:10.1371/journal.pntd.0010083)
Supplement: S1 Text — (PDF) [file pntd.0010083.s001.pdf]

S1 Text

Random walk instructions

In accordance with precedent [1], the team started from the communal center of each neighborhood, often an intersection of two roads, a shop, a church, or a meeting place and identified possible residential paths originating from the neighborhood center. A neighborhood skip number was calculated by dividing the sample population goal for that neighborhood by an estimate of the total number of households in that neighborhood. They randomly selected a path and visited households in a skip pattern determined by the neighborhood skip number. When the path forked, they would go down both forks. When the path ended, led to territory allocated to another of the original paths, or a neighborhood boundary was reached, they returned to the neighborhood center and selected a new path until the neighborhood quota was reached.

References

1. S. Bennett, T. Woods, W. Liyange, and D. Smith, "A simplified general method for cluster-sample surveys of health in developing countries," *World Health Statistics*, vol. 44.
