# Supplementary material for: Community trust of government and non-governmental organizations during the 2014-16 Ebola epidemic in Liberia
Source: PLoS Negl Trop Dis. 2022 Jan 27;16(1):e0010083. doi: 10.1371/journal.pntd.0010083 (PMC8824372; doi:10.1371/journal.pntd.0010083)
Supplement: S1 Table — (PDF) [file pntd.0010083.s002.pdf]

S1 Table

S1 Table. Social capital-related questions and answers for the sample population (n=1433).
These questions were adapted for Liberian context from the social capital resource generator questions developed by Van der Gaag and Snijders [1].

Do you know anyone who...	% yes	If yes, access through...			"And yourself?"
		acquaintance	friend	family member	
Can repair a car, bike, or other mechanical object	68	39	34	27	5.9
Owns a motorbike	83	25	44	3	7.8
Is handy repairing household equipment	60	38	34	29	9.6
Can speak and write a foreign language	37	33	38	28	6.0
Can work with a personal computer	52	18	44	39	9.1
Can play an instrument	66	45	35	20	9.1
Has knowledge of literature	23	36	39	25	8.7
Has senior high school education	97	3.4	17	79	-
Has higher than senior high school education	89	5.8	20	74	-
Owns a television	75	15	32	53	22
Is active in a political party	51	34	40	26	18
Works in the government	53	23	24	53	-
Owns a business	89	17	40	43	38
Is sometimes in the opportunity to hire people	42	41	34	24	13
Knows a lot about governmental regulations	33	31	28	41	6.2
Has good contacts with a newspaper, radio, or TV station	36	38	42	21	6.8
Knows about soccer	90	12	44	45	35
Has knowledge about financial matters (taxes, etc.)	32	26	32	42	13
Can find a temporary job for a family member	34	23	29	48	-
Can give advice concerning conflict with others	94	9.3	22	6.9	-
Can help with lifting, packing, or heavy work	64	25	39	36	-
Can go to the market for you when you are ill	95	1.6	25	74	-
Can give medical advice if you need it	70	16	33	52	-
Can lend you a large sum of money	24	12	40	48	-
Can provide a place to stay for a week	84	1.7	23	75	-
Can give advice concerning a conflict with family	97	4.8	20	76	-
Can discuss what political party you are going to vote for	55	29	43	28	-
Can give advice on matters of law	39	26	33	41	-
Can give a reference if you are looking for work	41	20	38	42	-
You can pay social visits too	92	0.8	45	54	-
Can discuss intimate matters with you	92	0.8	47	52	-
Can do small jobs around the house	80	12	20	68	-
Owns a smartphone	82	4.2	26	70	-

References

1. M. Van Der Gaag and T. A. Snijders, "The resource generator: social capital quantification with concrete items," *Social Networks*, vol. 27, no. 1, pp. 1–29, 2005.
