# Supplementary material for: Community trust of government and non-governmental organizations during the 2014-16 Ebola epidemic in Liberia
Source: PLoS Negl Trop Dis. 2022 Jan 27;16(1):e0010083. doi: 10.1371/journal.pntd.0010083 (PMC8824372; doi:10.1371/journal.pntd.0010083)
Supplement: S2 Table — (PDF) [file pntd.0010083.s003.pdf]

S2 Table

S2 Table. Generalized linear model regression coefficient estimates for trust in government and trust in iNGOs as dependent variables in time period 3 using an ordinal regression model (as in Fig 3). Some independent variables were dropped via AIC stepwise reduction from one or both models. A negative coefficient indicates a negative association with trust, while a positive coefficient indicates a positive association with trust. Community descriptions: Careysburg = rural, low exposure; Tubmanburg = urban, high incidence.

	**Government**			**iNGO**		
Independent variable	Value	Std Err	t value	Value	Std Err	t value
Careysburg	0.29	0.13	2.15	0.42	0.14	2.92
Tubmanburg	0.74	0.14	5.44	1.20	0.16	7.55
Knew someone infected	-0.25	0.11	-2.26	-0.28	0.13	-2.23
Belief in Ebola	0.71	0.16	4.59	0.62	0.17	3.70
Age	-0.11	0.05	-2.09	-	-	-
Frequent witness of Ebola	-	-	-	0.26	0.13	1.96
Social capital	-0.15	0.06	-2.71	-0.19	0.06	-3.03
Ebola knowledge	0.15	0.05	2.92	0.30	0.06	5.20
Highly mobile	-0.21	0.11	-1.94	-	-	-
